# Annotations

**Published:** 1904-04-09

**Authors:** 


### ANNOTATIONS.

### Hypnotism in Chronic Alcoholism.

In a recent article Dr. Lloyd Tuckey quotes statistics from various sources showing the success of hypnotism in the treatment of chronic alcoholism. His own practice gives the number of cases treated as 93 with 75 cures. Milne Bramwell shows 76 cases with 28 cures (17 men and 11 women). Both observers record instances of "benefit" in addition to those registered as "cured." Tokarsky, of Moscow, states that he has treated more than 700 patients, including representatives from all classes of the community, and claims to have cured 80 per cent. of those who wished to be cured and submitted themselves voluntarily to his treatment. He finds 15 to 20 hypnotic suggestions to be generally sufficient, but keeps the patient under observation for a year and does not reckon the case a "cure" until at least twelve mouths have passed without relapse. De Jong, of the Hague, has treated 41 drunkards in 13 years, and reports 19 of them as cured; in some instances the cure has been illustrated by 10 years' abstinence. Dr. Tuckey urges that alcoholic subjects ought to be given the chance of hypnotic treatment and points out the advantage this method possesses over confinement in a retreat. He finds that most alcoholics are good hypnotic subjects and places considerable stress upon the desire of the patient to be cured as an important element in successful treatment. It matters little what method of hypnotisation is adopted, and any doctor who has the confidence of his patient can practise the treatment with a reasonable prospect of success provided both practitioner and patient take the matter seriously. The suggestions should aim not merely at creating a negative or repellent feeling towards alcohol, but should also propose to restore self-control. It is possible to secure the first and yet to fail in the great object of the treatment. Dr. Tuckey protests against the statement that women drunkards are incurable. He finds, on the contrary, that their chances of cure are at least equal to those of men. With a view to increase the security of the value of hypnotic treatment it would be well that all those who have tried it should record their experiences.

### British Health Resorts.

The development of the home market has been strongly pressed upon the attention of the British public during the last few months, and it is quite in harmony with certain modern tendencies that an organised attempt should be made to proclaim the advantages of British health resorts. No one proposes to belittle the practical benefits associated with certain Continental spas, or to endeavour to depreciate the attractions of travel in new countries. All that is suggested is that steps shall be taken to secure the due presentation of the claims of the various places in our own country which offer opportunities to those in search of health or engaged in holiday enterprise. There can be no doubt that the local authorities at home have to a very large extent failed to recognise that if visitors are to be attracted business methods must be adopted and practised. In most places, fortunately, the old-fashioned opposition to modern sanitary reform has disappeared, and most of our home health resorts can now offer themselves without anxiety to the criticism of the most keen of health officials. Something more might often be done in the way of providing entertaiment for visitors, but there is an especial need that the advantages which exist shall be made known to the public, and that every facility shall be offered to those who are disposed to avail themselves of these advantages. At present, however, it is beyond the power of municipal authorities to advertise the merits of any health or holiday resort, without obtaining the special sanction of Parliament. But it would indeed be strange if this permission should continue to be withheld in face of the important developments that might be expected if the real merits of our own health resorts were to become more widely appreciated than they are at present. In the meantime it will be a distinct advance if different localities can be persuaded to combine for the purpose of announcing their attractions. The more widely the advantages of the home health resorts are known the better it will be for all of them, though petty local jealousies sometimes obscure this truth.

### Post-mortem Examinations in Hospitals.

It may be taken as an axiom that careful pathological examinations of patients who have died in a hospital are absolutely essential, both in the interests of the public and for the advancement of the science and art of healing. It is hardly too much to say that the reputations of surgeons and physicians are made or lost in the post-mortem room. Here it is that diagnostic acumen is put to the surest test, and here the mistakes of treatment, whether by operation or otherwise, are made manifest and beyond concealment by the hardiest bluff. In view of such considerations we do not suppose that any intelligent person will be found to plead that autopsies are unnecessary and objectionable. Whether such absence of opposition would continue if this country were to follow the lead of some others and make post-mortem examinations of patients dying in public hospitals compulsory by law is questionable. Rather we should expect the "anti" folk to arise and commence agitating for the abolition of autopsies altogether. A Bill has recently been introduced into the New York legislature to render post-mortem examinations of patients dying in a public hospital compulsory. If this Bill becomes law we shall follow its effects with considerable interest. Certainly in this country under existing arrangements there is not usually much difficulty in obtaining the necessary permission from the relatives or friends of the deceased when the necessities of the case are clearly explained to them. This is so much to the credit of the average individual; for the mutilation of the dead is a matter to which sentiment is entirely opposed. Whether such intelligence would be exercised in opposition to the mandates of the professional agitator may well be doubted.

